# Propofol Affords No Protection against Delayed Cerebral Ischemia in a Mouse Model of Subarachnoid Hemorrhage

**DOI:** 10.3390/diseases11040130

**Published:** 2023-09-27

**Authors:** Meizi Liu, Keshav Jayaraman, James W. Nelson, Jogender Mehla, Deepti Diwan, Ananth K. Vellimana, Gregory J. Zipfel, Umeshkumar Athiraman

**Affiliations:** 1 Molecular Cell Biology, Washington University, St. Louis, MO 63110, USA; 2 Department of Neurological Surgery, Washington University, St. Louis, MO 63110, USA; 3 Department of Radiology, Washington University, St. Louis, MO 63110, USA; 4 Department of Neurology, Washington University, St. Louis, MO 63110, USA; 5 Department of Anesthesiology, Washington University, Campus Box 8054, 660 South Euclid Avenue, St. Louis, MO 63110, USA

**Keywords:** propofol, aneurysmal subarachnoid hemorrhage, delayed cerebral ischemia, neurovascular protection

## Abstract

Delayed cerebral ischemia (DCI) is an important contributor to poor outcomes in aneurysmal subarachnoid hemorrhage (SAH) patients. We previously showed that volatile anesthetics such as isoflurane, sevoflurane and desflurane provided robust protection against SAH-induced DCI, but the impact of a more commonly used intravenous anesthetic agent, propofol, is not known. The goal of our current study is to examine the neurovascular protective effects of propofol on SAH-induced DCI. Twelve-week-old male wild-type mice were utilized for the study. Mice underwent endovascular perforation SAH or sham surgery followed one hour later by propofol infusion through the internal jugular vein (2 mg/kg/min continuous intravenous infusion). Large artery vasospasm was assessed three days after SAH. Neurological outcome assessment was performed at baseline and then daily until animal sacrifice. Statistical analysis was performed via one-way ANOVA and two-way repeated measures ANOVA followed by the Newman–Keuls multiple comparison test with significance set at *p* < 0.05. Intravenous propofol did not provide any protection against large artery vasospasm or sensory–motor neurological deficits induced by SAH. Our data show that propofol did not afford significant protection against SAH-induced DCI. These results are consistent with recent clinical studies that suggest that the neurovascular protection afforded by anesthetic conditioning is critically dependent on the class of anesthetic agent.

## 1. Introduction

Aneurysmal subarachnoid hemorrhage (SAH) is a type of hemorrhagic stroke with very high morbidity and mortality [1]. The amount of bleeding in the brain and the secondary brain injury caused by the bleeding are the two most important determinants of patient outcomes after SAH. The major treatable cause of secondary neurological injury in these patient populations is delayed cerebral ischemia (DCI) [1]. DCI occurs in 30% of patients, 4–12 days after SAH, and is characterized by large artery vasospasm and microcirculatory deficits [2,3]. Numerous therapies for DCI have been tried so far without success, probably due to centering the treatments on a single element of what is now known to be a multifaceted process. To overcome this issue, we and others applied a therapeutic strategy called conditioning—a powerful and extremely pleiotropic neuroprotective strategy known to provide protection against several central nervous system cell types such as neurons, glia, and vascular cells [4]. In recent years, structurally distinct conditioning agents (e.g., anesthetics, hypoxia) have been shown to provide strong DCI protection leading to improved neurologic outcomes after SAH [2,3,5].

In our previous preclinical studies, we demonstrated that conditioning with volatile anesthetics, specifically isoflurane, provides robust multifaceted protection against SAH-induced DCI including large artery vasospasm, microvessel thrombosis, and short-term neurological deficits [2,3]. Our follow-up study showed that clinically relevant doses of commonly used volatile anesthetics such as sevoflurane and desflurane also afforded significant neurovascular protection against SAH-induced DCI, leading to improved neurobehavioral outcomes [6]. Supportive of our preclinical studies, our observational clinical studies showed that in SAH patients, receiving volatile anesthesia alone (sevoflurane or desflurane) for aneurysm treatment (clipping/coiling) was associated with lower incidence of angiographic vasospasm and DCI compared to SAH patients who received combined anesthesia (volatile anesthesia and propofol infusion) or only intravenous propofol anesthesia [7,8,9]. Propofol is a commonly used intravenous anesthetic agent in the SAH patient population, but at present, experimental studies examining the impact of propofol on SAH-induced DCI and neurological deficits are lacking. Therefore, the aim of our current study is to examine the effects of intravenous propofol on SAH-induced DCI.

## 2. Materials and Methods

**Figure 1:**
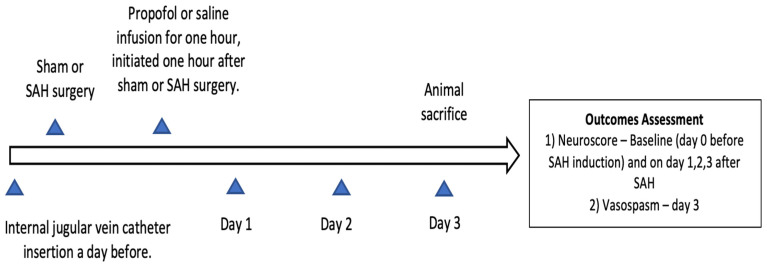
Experimental design of the study. SAH—subarachnoid hemorrhage.

Approval to conduct this study was obtained from the institutional animal care and use committee of Washington University in Saint Louis (Protocol no. 20180080, Approval date 22 July 2019) and it was confirmed to follow the National Institutes of Health Guidelines for the Care and Use of Animals in Research. Twelve-week-old wild-type male mice (C57BL/6) were obtained from Jackson laboratories (Bar Harbor, ME, Strain #:000664) for the study. Mice were placed in temperature- and humidity-controlled rooms with a 12 h dark–light cycle. Five mice were housed in a cage with free access to laboratory chow and water. The experimental groups were divided into sham (*n* = 14), SAH (*n* = 13), and SAH + propofol conditioning (*n* = 16) groups. Mice were randomly assigned to one of the above-mentioned experimental groups and the experiments were replicated independently (a minimum of three times) with all three groups represented on each day. Neuroscore and large artery vasospasm assessment and data analysis were conducted in a blinded manner. The endovascular perforation SAH model and the internal jugular vein catheter insertion in the mice were performed by an experienced technician from our laboratory. All mice that underwent SAH had a brief episode of apnea, and none of the sham mice experienced apnea. SAH induction was confirmed by the apneic episode after endovascular perforation and by the identification of blood on the ventral surface of the brain after animal sacrifice. Animals which died during or after the SAH procedure, and mice with improper staining or an unclear middle cerebral artery vessel, were not included in the analysis; the rest of the animals were included. Surgical procedures and outcome assessments were performed during the light phase of the 12 h dark–light cycle. The overall design of the experiment is represented in Figure 1. We followed the Animal Research: Reporting of In Vivo Experiments guidelines for this study.

### 2.1. Mouse Endovascular Perforation SAH Model

Endovascular perforation SAH was performed in mice per our previously published methods [2,3,6]. Briefly, isoflurane (4% induction, 1.5% maintenance) in room air was utilized to anesthetize mice. Normothermia at 37 °C was maintained throughout the procedure using a thermo-regulated heating pad (mTCII micro-Temperature Controller’ by Cell Micro Controls, Norfolk, VA, USA, Accuracy: ±0.2 °C). Following antiseptic precautions, a midline incision was made in the neck and the external carotid artery (ECA) was exposed. A 5–0 nylon suture was introduced via ECA and advanced distally through the internal carotid artery until resistance was felt at the bifurcation of anterior cerebral artery and middle cerebral artery (MCA). In the SAH groups, the suture was advanced further to induce SAH, and then removed, and the ECA was ligated. The sham mice underwent similar steps except that the suture was removed without causing perforation. Isoflurane duration for sham or SAH surgery was short and consistent across groups. Post sham or SAH procedure, the mice were recovered in a heated incubator before returning to their corresponding cages.

### 2.2. Propofol Conditioning

To facilitate intravenous (IV) propofol or saline infusion, a central line catheter was inserted through the left internal jugular vein in all groups during the brief period of isoflurane anesthesia. Propofol exposure was achieved by administering an IV propofol infusion (2 mg/kg/min for one hour) beginning one hour after SAH using an automatic injector (Stoelting, Wood Dale, IL, USA) with the mice placed inside a mouse restrainer. To maintain normothermia during propofol infusion, the mouse restrainer was placed above a homeothermic blanket (HTP-1500 heat therapy pump, Kent Scientific Corporation, Torrington, CT, USA, Accuracy: ±2 °F). Propofol conditioning or saline infusion was instituted twenty-four hours post central line catheter insertion. Propofol dosing in the current study was chosen to maintain a constant brain propofol concentration, producing a steady state of general anesthesia [10].

### 2.3. Cerebral Vasospasm Measurement

Middle cerebral artery (MCA) diameter, as a measure of vasospasm, was examined on day 3 after SAH as per our published methods [2,3,6]. Briefly, mice were anesthetized with isoflurane and a pressure-controlled cerebrovascular casting was performed with phosphate buffered saline, 10% formalin and ROX SE (5-(and-6)-Carboxy-X-rhodamine, succinimidyl ester) at 72 h post sham or SAH surgery. Brains were then extracted and blood vessels in the circle of Willis were imaged under a fluorescent microscope using a CCD camera (CoolSNAP EZ, Photometrics, Tucson, AZ, USA) and MetaMorph^®^ software (Universal Imaging, West Chester, PA, USA). Cerebral vasospasm was measured in the left (ipsilateral) MCA. The average of three independent measures of the smallest width in the first 1 mm segment of the MCA from internal carotid artery bifurcation was calculated as a measure of vasospasm.

### 2.4. Neurobehavioral Assessment

Neurological outcome was evaluated based on our previously published methods [2,3,6]. Briefly, neurological function was graded based on a motor score (0 to 12) and a sensory score (4 to 12). Components examined for motor score were spontaneous activity, symmetry of limb movements, climbing, and balance and coordination. Components examined for sensory score were body proprioception, vibrissa, visual, and tactile responses. Neurobehavioral testing was performed on day 0 right before SAH induction, and on days 1, 2, and 3 until the animals were sacrificed. The total neuroscore ranges from 4–24, with higher numbers representing better neurologic outcomes.

### 2.5. Statistical Analysis

Statistical analysis was performed using Prism software (GraphPad software, version 9.0.0, La Jolla, CA, USA). Data are expressed as mean ± standard error of mean. Large artery vasospasm was evaluated using one-way ANOVA followed by the Student Newman–Keuls multiple comparison test. Neurological outcomes were analyzed using two-way repeated measures ANOVA followed by the Student Newman–Keuls multiple comparison test. Statistical significance was fixed at *p* < 0.05.

## 3. Results

### 3.1. Propofol Conditioning Did Not Afford Protection against SAH-Induced Large Artery Vasospasm in Wild-Type Mice

**Figure 2:**
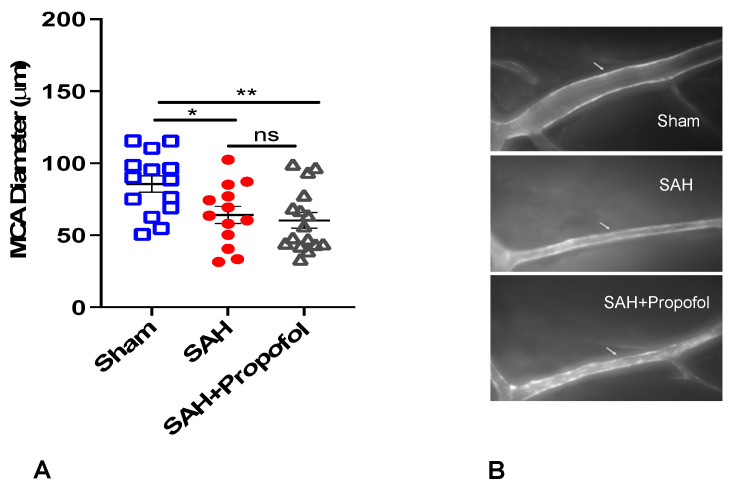
Propofol did not attenuate vasospasm induced by SAH in an endovascular perforation mouse model. Wild-type male mice (WT) underwent SAH or sham surgery followed 1 h later by intravenous propofol infusion (2 mg/kg/min) for 1 h. Vasospasm was assessed on day 3 (**A**). (**B**) Representative images for vasospasm. The arrow mark points to the ipsilateral left middle cerebral artery (MCA). Data indicate mean ± SEM. (**A**) * *p* < 0.05 sham vs. SAH, sham vs. SAH + Propofol, (ns), *p* > 0.05 SAH vs. SAH + Propofol; ANOVA followed by Student Newman–Keuls comparison test. ns—nonsignificant. SEM—standard error of mean. SAH—subarachnoid hemorrhage. * *p* < 0.05, ** *p* < 0.01.

A total of 55 wild-type mice were used for the experiment. Out of 55 mice, 9 died in SAH groups and 3 were excluded as the MCA vessels were not clearly stained. Therefore, our final analysis included a total of 43 mice with *n* = 14 in the sham group, *n* = 13 in the SAH group and *n* = 16 in the SAH + propofol group. All animals in the SAH group experienced apnea immediately after perforation, but none in the sham group developed apnea. Mice in the SAH group showed significant vasospasm compared to the sham group (*p* < 0.05, Figure 2). Administration of propofol did not afford protection against SAH-induced large artery vasospasm (*p* > 0.05, Figure 2A,B).

### 3.2. Propofol Conditioning Did Not Afford Protection against SAH-Induced Neurological Deficits in Wild-Type Mice

**Figure 3:**
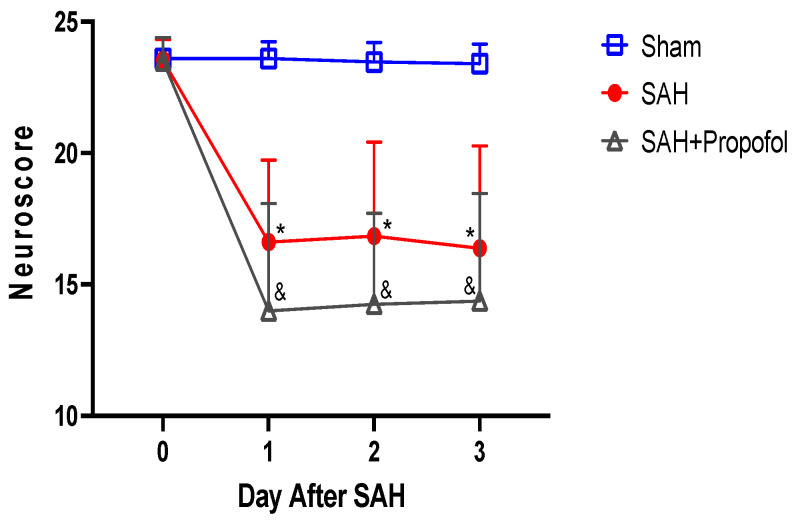
Propofol did not improve neurologic outcomes after SAH in an endovascular perforation mouse model. Wild-type male mice (WT) underwent SAH or sham surgery followed 1 h later by intravenous propofol infusion (2 mg/kg/min) for 1 h. Neuroscore was assessed baseline and daily for three days after SAH. Data indicate mean ± SEM. * & *p* < 0.05 sham vs. SAH, sham vs. SAH + Propofol, * & *p* < 0.05 SAH vs. SAH + Propofol, by two-way repeated measures ANOVA followed by Student Newman-Keuls comparison test. SEM—standard error of mean. SAH—subarachnoid hemorrhage.

Mice in the SAH group showed significant neurologic deficits compared to the sham group (*p* < 0.05, Figure 3). Administration of propofol did not afford protection against SAH-induced neurologic deficits (*p* > 0.05, Figure 3).

## 4. Discussion

The main findings in our study are that the intravenous anesthetic propofol does not provide protection against either large artery vasospasm or neurologic deficits induced in a common mouse model of SAH. The present preclinical data confirm our previous finding in SAH patients that intravenous propofol does not protect against angiographic vasospasm or DCI. These findings are critical, as SAH patients are commonly exposed to anesthetics during their early ictus period for diagnostic purposes (i.e., catheter angiography), aneurysm treatment (clipping/coiling), and/or sedation while in the intensive care unit. Hence, the selection of appropriate anesthetics during this critical period may significantly impact patient outcomes.

### 4.1. Propofol against SAH-Induced DCI

Propofol is a commonly used intravenous anesthetic agent for induction and maintenance in the operating room for aneurysmal repair procedures (coiling/clipping), and also one of the commonly used anesthetics in the intensive care unit for sedating mechanically ventilated SAH patients. However, studies examining the impact of propofol on SAH-induced DCI are lacking. A preliminary clinical study in SAH patients undergoing aneurysmal clipping under propofol anesthesia measured the plasma concentrations of endothelin-1(ET-1) and calcitonin gene related peptide (CGRP) and showed that intravenous propofol anesthesia significantly reduced CGRP levels, but had no effect on ET-1 levels. Given the fact that CGRP is a significant vasodilator, the authors speculated that intravenous propofol may play a role on the pathogenesis of cerebral vasospasm in SAH patients [11]. Another clinical study noted that SAH patients who received propofol as the primary anesthetic for an intracranial aneurysm repair procedure had an increased incidence of transcranial doppler evident vasospasm compared to patients who received desflurane for the procedure [12]. However, no significant differences were noted between the two anesthetic groups in relation to other outcomes such as angiographic vasospasm, cerebral infarction, or clinical outcomes as measured using the Glasgow Coma Scale score on day 14 after surgery, and the Glasgow Outcome Scale score at 3 months.

Complementing the previous studies, our series of clinical studies suggested that volatile anesthetics have a robust neuroprotective effect against cerebral vasospasm and DCI in SAH patients compared to intravenous propofol [7,8,9]. In a small cohort of SAH patients (157) undergoing aneurysm repair (clipping/coiling), we showed that SAH patients who received volatile anesthetics (sevoflurane or desflurane) for anesthetic maintenance had a lower incidence of angiographic vasospasm compared to SAH patients who received combined anesthetics (sevoflurane or desflurane and intravenous propofol) [7]. In a follow-up study with a larger patient cohort (390), we provided additional evidence showing that SAH patients receiving volatile anesthetics (sevoflurane or desflurane) had less angiographic vasospasm and DCI [8]. Direct evidence came from our recent study where we compared SAH patients receiving volatile anesthetics (sevoflurane or desflurane) during aneurysm repair at one academic institution to SAH patients who received only intravenous anesthesia (propofol) at a different academic institution. We noticed that SAH patients exposed to volatile anesthetics were less likely to develop angiographic vasospasm and DCI compared to SAH patients who received propofol [9]. However, no significant difference was noted between the groups in functional outcomes at patient discharge as measured via the modified Rankin scale or discharge disposition. To the best of our knowledge, the present study is the first experimental study examining the effects of propofol on large artery vasospasm and neurologic outcome in a murine SAH model. Supporting the clinical findings from our group and others, we did not find that propofol provides any protection against large artery vasospasm or the neurological deficits induced by SAH in a mouse model of SAH.

### 4.2. Volatile vs. Intravenous Anesthetics for SAH-Induced DCI

The potential reasons for the neuroprotective effect of commonly used halogenated volatile anesthetics (isoflurane, sevoflurane, desflurane) compared to intravenous propofol in SAH-induced DCI are the following. (1) Volatile anesthetics are shown to have a direct effect on cerebral vasculature, causing vasodilation and resulting in a dose-dependent increase in cerebral blood flow, whereas intravenous propofol was shown to significantly reduce cerebral blood flow, possibly due to maintaining an intact flow–metabolism coupling in the cerebral vasculature [13,14,15]. (2) An earlier experimental study elucidating the mechanism of volatile anesthetic conditioning-induced protection in DCI was attributed to the upregulation of hypoxia inducible factor—1 alpha (HIF-1α) [16]. HIF-1α is a transcriptional factor involved in regulating multiple genes that are known to affect cerebral vessel function [17,18]. Though no experimental studies have yet evaluated the impact of propofol on SAH-induced DCI, it has been shown that propofol inhibits HIF-1α activation [19]. Another mechanism by which volatile anesthetics may provide cerebral vessel protection is through downregulating the potent vasoconstrictor ET-1. An in vitro study by Park et al. demonstrated that isoflurane significantly reduced cortical microvessel vasoconstriction induced by ET-1 in a mouse model of SAH [20]. Interestingly, a small clinical study in SAH patients undergoing aneurysm clipping showed that the volatile anesthetic desflurane, used during the maintenance period, significantly reduced plasma concentrations of ET-1, suggesting that desflurane may potentially reduce acute cerebral vasospasm in SAH patients [21]. The same group demonstrated that anesthetic maintenance with propofol did not decrease plasma ET-1 concentration in SAH patients undergoing aneurysm clipping [11]. (3) It is also possible that the two classes of anesthetic agents cause longer-term differential effects on the cerebrovasculature. For example, propofol has been shown to exacerbate vascular smooth cell and endothelial cell injury resulting in vascular dysfunction [22,23,24], while isoflurane has an opposite effect [25,26,27].

### 4.3. Clinical Benefits of Volatile Anesthetics in SAH Patients

We previously demonstrated that clinically relevant doses of commonly used volatile anesthetics such as isoflurane, sevoflurane and desflurane afforded strong protection against SAH-induced DCI, including improved short-term neurological outcomes [2,3,6]. These findings are supplemented by our observational studies showing that volatile anesthetics are associated with a lower incidence of angiographic vasospasm and DCI compared to intravenous propofol [7,8,9]. Validation of these findings in prospective clinical trials will have a significant clinical impact in several ways. (1) It could provide guidance for optimizing the anesthetic management of SAH patients undergoing various intraoperative and neuro-interventional procedures that could ultimately improve patient outcomes. (2) Recent studies have shown that volatile sedation is a safe alternative to intravenous sedation in ICU patients [28,29,30,31]. Given the fact that a subanesthetic dose (patient is sedated but not anesthetized) of isoflurane affords robust neurovascular protection against SAH-induced DCI [32], it is conceivable that volatile anesthetics could replace intravenous sedation in the ICU for SAH patients. (3) Molecular therapies could be designed to mimic the protection provided by volatile anesthetics and could be developed as a stand-alone therapeutic strategy as a means for reducing secondary brain injury and improving neurological outcomes in SAH patients.

### 4.4. Strengths and Limitations

The strengths of our study are: (1) This is the first experimental study to explore the impact of propofol on SAH-induced DCI. This information is critical to know, as practitioners commonly utilize only intravenous anesthesia (propofol) for the management of SAH patients for various interventions. (2) We simulated the administration of propofol in human patients by running a continuous intravenous propofol infusion through a central line catheter in the mice. An alternative option to run a continuous propofol infusion in mice is via the tail vein; however, this route of administration, for prolonged infusions, has been shown to be extremely unreliable [10]. Our study is not without limitations. (1) Though our current mouse model of SAH reflects its features in humans, alternate animal models of SAH should be used to confirm our findings. (2) Only male mice were utilized in the current experiments. Future studies should include both genders to examine the impact of propofol conditioning on SAH-induced DCI and neurologic outcomes. Finally, (3) the impact of propofol conditioning on long-term neurobehavioral outcomes after SAH was not examined. This information is essential for future translational studies.

## 5. Conclusions

Our data show that propofol, a commonly used intravenous anesthetic in SAH patients, does not afford protection against large artery vasospasm or neurological deficits produced by SAH. Further prospective randomized clinical studies are warranted to examine the effects of volatile anesthetics and intravenous propofol on secondary brain injury and neurologic outcomes in SAH patients.

## Data Availability

All data in the study are available by a reasonable request to the corresponding author, Umeshkumar Athiraman (uathira@wustl.edu).
